# Different cutoff values of the skeletal muscle mass and myosteatosis result in different clinical impact on overall survival in oncology. A subanalysis of a clinical trial

**DOI:** 10.1007/s00432-025-06190-1

**Published:** 2025-04-16

**Authors:** Alexey Surov, Maximilian Thormann, Andreas Wienke, Jens Ricke, Max Seidensticker

**Affiliations:** 1 Department of Radiology, Neuroradiology and Nuclear Medicine, Johannes Wesling University Hospital, Ruhr University Bochum, Bochum, Germany; 2 Department of Nuclear Medicine, Charité Berlin, Augustenburger Platz 1, 13353 Berlin, Germany; 3 Department of Radiology, University Hospital Magdeburg, Magdeburg, Germany; 4 Institute of Medical Epidemiology, Biometry and Informatics, University of Halle, Halle, Germany; 5 Department of Radiology, University Hospital, LMU Munich, Munich, Germany

**Keywords:** Sarcopenia, Myosteatosis, Skeletal Muscle Index (SMI), Muscle attenuation, Prognosis

## Abstract

**Background:**

Body composition analysis, particularly the assessment of sarcopenia and myosteatosis, has emerged as a potential prognostic tool in oncology. However, the clinical implication of body composition parameters remains inconsistent, largely due to the variability in cutoff values used across studies. This study examines the influence on prevalence and prognostic influence of different cutoff values for sarcopenia and myosteatosis in patients in a standardized cohort from a large clinical trial (SORAMIC).

**Methods:**

This study included 179 patients with unresectable liver cancer from the palliative arm of the SORAMIC trial. Skeletal muscle index (SMI) was calculated by measuring the cross-sectional area of skeletal muscle at the third lumbar vertebra (L3) on baseline CT scans. We then applied 14 published cutoff definitions for sarcopenia (SMI) and 7 for myosteatosis (muscle attenuation) to determine their prevalence in this cohort. Cox regression models were used to analyze the relationship between sarcopenia, myosteatosis, and OS.

**Results:**

The prevalence of sarcopenia ranged from 8.9% (Van der Werf et al.) to 69.8% (Lanic et al.). Overall, 3 of the 14 cutoffs [Van Vledder et al. (HR = 1.53, p = 0.03), Coelen et al. (HR = 1.46, p = 0.03), and Derstine et al. (HR = 1.47, p = 0.04)] showed a relevant association with OS. Other cut off values were not associated with OS.

The prevalence of myosteatosis varied between 10.1% (Nachit et al.) and 53.1% (Zhang et al.). One of the 7 cutoffs (Chu et al.) demonstrated a relevant association with OS (HR = 1.53, p = 0.03).

**Conclusion:**

The large variability in prevalence and prognostic impact observed across different cutoff definitions underscores the urgent need for standardized, cancer-specific cutoff values for SMI and muscle attenuation. Establishing uniform criteria will enhance the reliability and clinical applicability of body composition metrics as prognostic tools in oncology. Further research should focus on refining these cutoffs and validating them across diverse cancer populations.

## Introduction

Body composition analysis, particularly the assessment of skeletal muscle mass (SMM), and intramuscular fat, has emerged as a potential prognostic tool in oncologic diseases. Low skeletal muscle mass (LSMM), an image-based proxy for sarcopenia, is particularly prevalent in patients with malignant diseases and has been associated with increased morbidity and mortality (Rier et al. 2016; Surov and Wienke 2022; Simonsen et al. 2018). The muscle area determined based on the computed tomography (CT) at the level of L3 correlates well with the total body muscle mass (Prado et al. 2008). In patients with cancer, LSMM has been shown to be prognostic for overall survival (OS) and treatment response (Surov et al. 2023a, 2021, 2023b).

Another readily obtainable metric from CT imaging is skeletal muscle attenuation (MA) (Goodpaster et al. 2000). MA offers valuable insight into fat infiltration within muscle fibers—myosteatosis—which is indicative of reduced muscle quality (Rahemi et al. 2015). In patients with cancer, myosteatosis was prognostic for worse OS (Aleixo et al. 2020; Bin and Liang 2023).

Measurements of muscle mass and muscle attenuation in patients with cancer can be easily performed on routine staging CT, providing information not only about muscle quantity but also quality (Williams et al. 2021). However, there is still no consensus on standardized CT-derived cut-off values for low skeletal muscle mass and myosteatosis. Although various cutoff values exist for low muscle mass and low muscle density, there are wide discrepancies between the proposed cutoff values. Values are derived from different study populations and with varying techniques and scanning parameters. This lack of agreement hinders accurate data analysis, interpretation, and its translation into clinical practice (Westenberg et al. 2022). The cutoffs used in various studies are often derived from reference populations with different demographic and clinical characteristics, leading to potentially misleading conclusions when applied universally (Wang et al. 2023).

Patients from a large standardized clinical trial provide a good opportunity to assess the impact of different reference and cutoff values of sarcopenia and myosteatosis on clinical parameters such as OS. In this study, our aim was to explore how different cutoff values affect the prevalence of sarcopenia and myosteatosis in a cohort from a clinical trial and their impact on OS.

## Materials and methods

### Patients

For the present analysis, a standardized cohort from a large clinical trial was selected. This was the palliative arm of the SORAMIC trial (NCT01556490), a prospective, randomized controlled, phase II trial that enrolled patients with unresectable hepatocellular carcinoma (HCC) at 38 clinical sites in 12 countries in Europe and Turkey. The study design and procedural details have been reported elsewhere (Ricke et al. 2019).

In brief, patients were eligible for the SORAMIC trial if they had preserved liver function (Child-Pugh score ≤ B7), an Eastern Cooperative Oncology Group performance status (ECOG PS) ≤ 2, and unresectable HCC not eligible for curative treatment or transarterial chemoembolization. Patients in the palliative part of the study were randomized to receive either sorafenib monotherapy or SIRT and sorafenib. All patients provided written informed consent to participate in the study (ClinicalTrials.gov No. NCT01126645; EudraCT 2009-012576-27).

For this substudy, all patients from the sorafenib arm of the palliative part of SORAMIC were selected. No patients were excluded. Out of 179 patients in total, there were 24 women (13.4%) and 155 men (86.6%), with a mean age of 65.8 ± 8.9 years. Median OS was 11 months. Baseline patient characteristics are summarized in Table 1**.**

**Table 1:** Baseline patient characteristics

Characteristics	All patients (N = 179)
Age (mean, SD)	65.8 ± 8.9
Men/women (%)	155 (86.6 %)/24 (13.4 %)
Etiology	Alcohol: 64 (35.8%) HCV: 33 (18.4%) Cryptogenic: 24 (13.4%) HBV: 17 (9.5%) NAFLD: 11 (6.2%) NASH: 11 (6.2%) Alcohol + viral: 10 (5.6%) Not specified: 4 (2.2%) Hemochromatosis: 3 (1.7%) Alpha 1 antitrypsin deficiency: 1 (0.6%) AIH: 1 (0.6%)
BMI (median, range)	26.2 kg/m^2^ (16.0–46.5 kg/m^2^)
Overall survival (median, range)	11 months (0.2–59.5 months)
SMI (median, range)	50 cm^2^/m^2^ (29.5–73.6 cm^2^/m^2^)
Muscle density (median, range)	39 HU (10.8–60.0 HU)

### Body composition analysis

**Figure 1.**
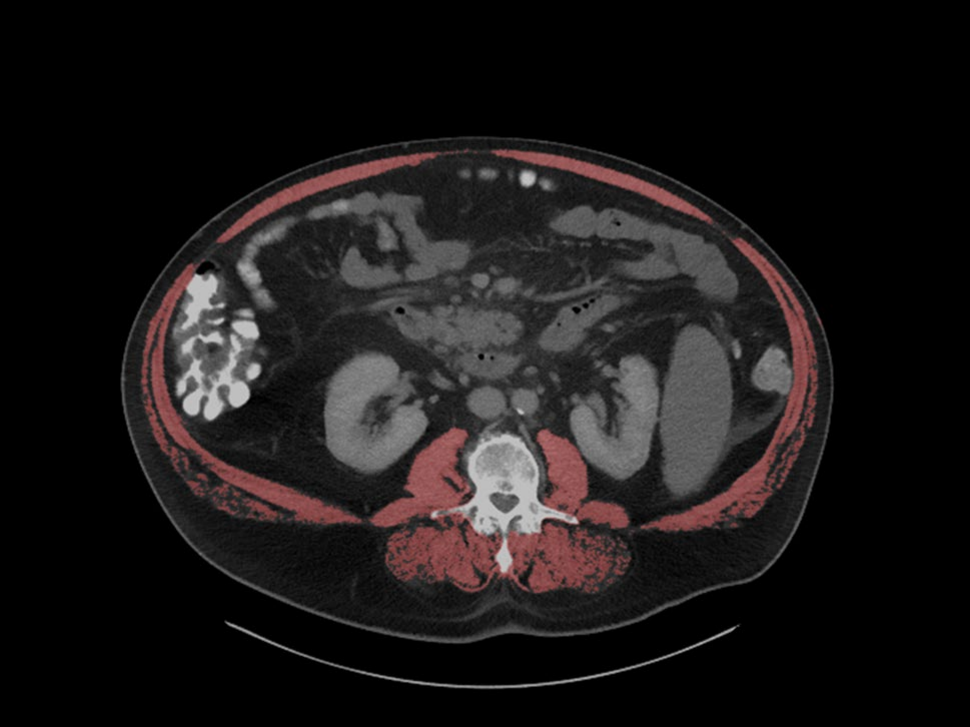
Segmentation of the skeletal musculature at the L3 level

For all patients, baseline CT scans, obtained prior to initiation of therapy, were utilized for analysis. CT imaging was performed with a standardized CT protocol in the portal venous phase (Ricke et al. 2019). All parameters of the skeletal musculature were measured in a semi-automatic fashion using axial images at the level of the third lumbar vertebra (L3) using standard Hounsfield unit ranges (− 29 to + 150 HU). ImageJ software (version 1.53, National Institute of Health, USA), a freely available imaging processing program, was employed for this purpose. To ensure accurate assessment, an experienced radiologist (AS), who remained blinded to clinical outcomes and treatments, corrected the software-based contours as needed before the measurements. Skeletal muscle area (SMA) was defined as the cross- sectional muscle area, including the quadratus lumbo-rum, psoas, rectus abdominis, and erector spinae muscles, and the internal transverse and external oblique muscles at the L3 level (Figure 1). Measurements of muscle tissue were normalized for patients’ body height in meters squared to attain the skeletal muscle index (SMI).

For MA, the HU values of the analyzed muscles were determined. The average HU value was noted.

Different thresholds of body composition analysis were applied to the images. The definitions for SMI are provided in Table 2, the definitions for myosteatosis (low MA) are provided in Table 3.

**Table 2:** Applied cut-off values for skeletal muscle index (SMI)

Author	Year	Cohort	Threshold
Prado et al. (2008)	2008	Solid tumours of the respiratory and gastrointestinal tracts	Male: < 52.4 cm^2^/m^2^ Female: < 38.5 cm^2^/m^2^
Baracos et al. (2010)	2010	Non-small cell lung cancer	Male: < 55.4 cm^2^/m^2^ Female: < 38.9 cm^2^/m^2^
Van Vledder et al. (2012)	2012	Hepatic surgery for CLM	Male: < 43.75 cm^2^/m^2^ Female: < 41.1 cm^2^/m^2^
Lanic et al. (2014)	2014	DLBCL	Male: < 55.8 cm^2^/m^2^ Female: < 38.9 cm^2^/m^2^
Fearon et al. (2011)	2011	International consensus	Male: < 55 cm^2^/m^2^ Female: < 39 cm^2^/m^2^
Carey et al. (2017)	2017	End stage liver disease	Male: < 50 cm^2^/m^2^ Female: < 39 cm^2^/m^2^
Coelen et al. (2015)	2015	Hepatectomy for perihilar cholangiocarcinoma	Male: < 46.8 cm^2^/m^2^ Female: < 39.1 cm^2^/m^2^
Derstine et al. (2018)	2018	Healthy US population	Male: 45.4 cm^2^/m^2^ Female: 34.4 cm^2^/m^2^
Van der Werf et al. (2018)	2018	Healthy caucasian population	Male: < 41.6 cm^2^/m^2^ Female: < 32.0 cm^2^/m^2^
Martin et al. (2013)	2013	Lung or gastrointestinal cancer	Male: < 53 cm^2^/m^2^for BMI ≥ 25; < 43 cm^2^/m^2^ for BMI < 25 Female: < 41 cm^2^/m^2^
Feliciano et al. (2017)	2017	Nonmetastatic colorectal cancer	Male: < 52 cm^2^/m^2^ for BMI < 30; < 54 cm^2^/m^2^ for BMI > 30 Female: < 38 cm^2^/m^2^ for BMI < 30; < 47 cm^2^/m^2^ for BMI > 30
Wendrich et al. (2017)	2017	Locally advanced head and neck cancer	< 43.2 cm^2^/m^2^
Caan et al. (2018)	2018	Nonmetastatic breast cancer	< 40 cm^2^/m^2^

*DLBCL* diffuse large B cell lymphoma, *US* united states, *CLM* colorectal liver metastases

**Table 3:** Applied cutoff values for muscle density

Author	Year	Cohort	Threshold
Molwitz et al. ( 2023)	2023	Patients with liver transplantation	< 38 HU
Martin et al. (2013)	2013	Lung or GI cancer	BMI < 25: < 41 HU BMI ≥ 25: < 33
Chu et al. (2020)	2020	Metastatic melanoma, ipilimumab-treated	BMI < 25 kg/m2: > < 4 2 HU BMI ≥ 25 kg/m2: < 20 HU
Sjoblom et al. (2016)	2016	NSCLC patients with disease stage IIIB/IV under first-line chemotherapy	male: < 28 HU female: < 23.8 HU
Zhang et al. (2023)	2023	Stage IIIB or IV NSCLC	male: < 40.11 HU female: < 33.59 HU
Nachit et al. (2023)	2023	Outpatients undergoing routine colorectal cancer screening	male: ≤ 18.9 HU female: ≤ 28.1 HU

### Statistical analysis

SPSS Version 25 and R were used for statistical analysis. Categorical data were reported by absolute and relative frequencies. To assess the impact of body composition values on clinical variables and OS, a Cox regression model was used. Hazard ratios are presented together with 95% confidence intervals (95% CI). Because of the exploratory nature of this re‐analysis of the data, all *p*‐values are interpreted in an exploratory sense.

## Results

### Sarcopenia

The prevalence of sarcopenia and the association with OS are given in Table 4.

**Table 4:** Prevalence of sarcopenia in the cohort for the different cut-off values and the results of the regression analysis for overall survival

Author	Prevalence of sarcopenia (%)	HR	95 % CI	p-value
Prado et al. (2008)	53.1	1.30	0.94–1.80	0.11
Baracos et al. (2010)	68.7	1.27	0.89–1.82	0.19
Van Vledder et al. (2012)	22.9	1.53	1.04–2.23	0.03
Lanic et al. (2014)	69.8	1.31	0.91–0.89	0.14
Fearon et al. (2011)	68.2	1.30	0.91–1.85	0.16
Carey et al. (2017)	45.8	1.37	0.99–1.90	0.06
Coelen et al. (2015)	32.4	1.46	1.04–2.06	0.03
Derstine et al. (2018)	24.0	1.47	1.01–2.14	0.04
Van der Werf et al. (2018)	8.9	1.31	0.74–2.32	0.36
Martin et al. (2013)	41.9	1.19	0.86–1.65	0.29
Feliciano et al. (2017)	53.1	1.22	0.88–1.69	0.23
Wendrich et al. (2017)	22.3	1.34	0.92–1.97	0.13
Caan et al. (2018)	14.0	1.43	0.90–2.27	0.13

The prevalence of sarcopenia differed substantially according to the cutoff definition applied. Of the 14 published SMI cutoff values evaluated, the lowest prevalence (8.9%) was observed with the definition by Van der Werf et al. (2018), and the highest prevalence (69.8%) was observed with the definition by Lanic et al. (2014).Three cutoff definitions [Van Vledder et al. (2012) Coelen et al. (2015) and Derstine et al. (2018)] demonstrated relevant associations with overall survival, with hazard ratios of 1.53 (95% CI 1.04–2.23, p = 0.03), 1.46 (95% CI 1.04–2.06, p = 0.03), and 1.47 (95% CI 1.01–2.14, p = 0.04), respectively. The remaining 11 cutoffs yielded hazard ratios ranging from 1.19 to 1.43, without reaching statistical significance (p values 0.06–0.36).

### Myosteatosis

The prevalence of myosteatosis and the association with OS are given in Table 5.

**Table 5:** Prevalence of myosteatosis in the cohort for the different cut-off values and the results of the regression analysis for overall survival

Author	Prevalence myosteatosis	HR	95 % CI	p-value
Molwitz et al. (2023)	45.8	1.12	0.81–1.55	0.48
Martin et al. (2013)	36.3	1.28	0.92–1.80	0.15
Chu et al. (2020)	21.8	1.53	1.04–2.26	0.03
Sjoblom et al. (2016)	11.7	0.76	0.44–1.29	0.30
Zhang et al. (2023)	53.1	0.98	0.71–1.36	0.92
Nachit et al. (2023)	10.1	0.92	0.53–1.62	0.78

Myosteatosis prevalence also varied considerably, from 10.1% (Nachit et al. 2023) to 53.1% (Zhang et al. 2023) across seven different muscle attenuation thresholds. One definition (Chu et al. 2020) showed a significant association with overall survival (hazard ratio 1.53, 95% CI 1.04–2.26, p = 0.03). The other six definitions produced hazard ratios between 0.76 and 1.28, none of which were statistically significant (p values 0.15–0.92).

## Discussion

A range of different cutoff values are proposed in the literature for the image-based diagnosis of sarcopenia and myosteatosis. In this study, we quantified the impact of the use of different cutoffs for the prevalence of sarcopenia and myosteatosis and their impact on OS in a standardized cohort from a large clinical trial. The variability in these findings emphasizes the urgent need for standardized cutoff values to ensure consistent and reliable prognoses in clinical practice.

Sarcopenia and myosteatosis are important prognostic parameters in clinical oncology (Surov et al. 2023b; Aleixo et al. 2020). However, CT-based criteria for sarcopenia and myosteatosis vary widely (Pickhardt 2022). There exists a large range of sarcopenia definitions in the oncological literature. The European Working Group on Sarcopenia (EWGSOP) and related consensus guidelines emphasize assessing muscle mass and quality, but they have not set any standard CT cutoff for sarcopenia in oncology (Cruz-Jentoft et al. 2019); Li et al. (2021). In our study we apply a selection of the most commonly used ones. Wang et al. showed that in 2020 alone there were over 50 new sarcopenia definitions added to the literature (Wang et al. 2023). Due to the work by Prado et al. (2008) and Martin et al. (2013) and the frequency of abdominal imaging in the oncological patient population, the assessment of muscle mass and quality increasingly focuses on image-based abdominal musculature. The earlier used Dual-energy x-ray absorptiometry is increasingly replaced by the measurement of muscle attenuation (Hounsfield units) and muscle area (adjusted for patient height as cm^2^/m^2^) using CT imaging as it is widely available in oncologic patients (Parkin and Renehan 2018). Original studies utilized optimum stratification to generate cutoff values for L3 SMI based on its prognostic ability for OS. The study by Prado et al., conducted with 2115 patients diagnosed with respiratory and gastrointestinal cancers, established sex-specific cutoff values for the L3 skeletal muscle index at 52.4 cm^2^/m^2^ for men and 38.5 cm^2^/m^2^ for women (Prado et al. 2008). An extension of this research, published by Martin et al., from the same research team in Edmonton, Alberta, Canada, refined these cutoff values by incorporating body mass index (Parkin and Renehan 2018). These cutoff points, however, have been subsequently applied indiscriminately, without consideration of tumor site, stage, or clinical variables. As a result, a patient’s sarcopenia status can change dramatically depending on which cut-off is applied. A recent meta-review of CT sarcopenia in pancreatic cancer found substantial variability between studies due to non-uniform measurement techniques and threshold values (Láinez Ramos-Bossini et al. 2024).

Our study applied various cutoff definitions for SMI and muscle density from the literature to a cohort from a large clinical trial. Notably, some cutoff definitions yielded a surprisingly low prevalence of sarcopenia and myosteatosis. For instance, using the muscle mass cutoff derived by Van der Werf et al. (2018) (based on a healthy population) identified only 8.9 % of our cohort as sarcopenic and the most restrictive myosteatosis criterion (Nachit et al. 2023) classified merely ~ 10% as having myosteatosis. Such low rates are in contrast to expectations in advanced HCC, where muscle wasting is common. This discrepancy suggests that cutoff values not tailored to HCC may underestimate the true burden of sarcopenia in this population. In fact, recent meta-analyses reported that 38.5%–42% of patients with HCC exhibit sarcopenia (March et al. 2022; Liu et al. 2023). In our trial cohort (which had relatively preserved performance status), many patients did not fall below certain high cutoffs, highlighting that inappropriate criteria can lead to misclassification of muscular status in HCC patients. Therefore, our findings reinforce that cancer-specific and context-specific cutoffs are needed for meaningful assessment of sarcopenia and myosteatosis in advanced HCC. Using definitions borrowed from other populations without validation in HCC may fail to identify a substantial subset of patients at risk, limiting the clinical utility of these biomarkers in guiding patient management.

A central novelty of our study is the systematic application of multiple published cutoff values—originating from diverse oncologic and non-oncologic populations—to a single advanced HCC cohort. This approach revealed how profoundly the choice of threshold influences prevalence and prognostic significance of sarcopenia and myosteatosis. We demonstrated that criteria drawn from different contexts lead to inconsistent correlations with survival. This finding is significant for advanced HCC because it highlights that the prognostic impact of sarcopenia or muscle quality can be easily missed or overestimated depending on how one defines these conditions. Our results therefore contradict previous analyses that found relevant associations with mortality across cancers for different definitions (Li et al. 2021).

Our study also exemplifies a broader issue: in the literature, many oncology studies have borrowed cutoffs from other tumor entities without validation. For example, of the 479 studies analyzed by Wang et al., only 162 referenced studies performed on the same tumor entity (Wang et al. 2023). Our results substantiate concerns that such practices introduce variability—only 2 of the definitions we applied were originally developed in HCC populations. For muscle density, the only cutoff showing relevant impact on OS in the sorafenib group was derived from a cohort of patients with malignant melanoma (Chu et al. 2020). This casts doubt on the clinical benefit of the current type of sarcopenia research. The significance in the context of HCC is clear: adopting a “one-size-fits-all” muscle index threshold from other cancers or healthy adults can be misleading.

Our findings put into perspective the results of prior studies on sarcopenia and myosteatosis in advanced HCC. Previous investigations have shown that low muscle mass and quality portend worse outcomes in HCC. For example, Antonelli et al. observed sarcopenia in approximately 49% of advanced HCC patients receiving sorafenib and noted that sarcopenic patients had significantly shorter overall survival (Antonelli et al. 2018). This prevalence is in line with the mid-range of values we observed when applying our multiple definitions (our range for sarcopenia prevalence spanned 8.9%–69.8%). Similarly, an Italian multicenter study by Biselli et al found roughly half of their advanced HCC cohort to be sarcopenic and confirmed an adverse impact on survival in both a training and validation set (Biselli et al. 2024).

Our work serves as evidence in support of harmonizing definitions. Our study contributes a necessary caution that comparisons across studies require consideration of the sarcopenia definition used, and it strengthens the call for standardized cutoffs. We believe this is an important step forward, as it provides a rationale for the development of consensus criteria in future HCC research, ultimately improving comparability and clinical translation of body composition findings.

The results of our analysis carry important clinical implications. They suggest that oncologists and radiologists should use caution when interpreting body composition metrics in HCC patients unless the cut-off values have been validated for that specific context. At present, there is no universal consensus on how to define CT-based sarcopenia or myosteatosis in HCC, and our study shows that this gap in standardization can directly alter clinical risk assessments. A solution could be to pool data from multiple HCC cohorts to determine an optimal skeletal muscle index and attenuation threshold that best stratifies outcomes in liver cancer patients. This will enhance the integration of body composition analysis into routine HCC care. Further prospective research should focus on validating these findings, exploring the mechanistic links between muscle depletion and liver cancer outcomes.

Significant challenges remain before standardization in measurement is achieved. These include population diversity, cancer type and stage (Thormann et al. 2025). A meta-analysis by Au et al. highlighted that the association between low lean mass and cancer mortality was not very strong in certain types of cancer (Au et al. 2021). A cancer-type specific approach is likely to yield more robust and accurate cutoff values, thereby improving our understanding of the prognostic implications of sarcopenia across different malignancies. Any consensus cut-off will need to be paired with standardized protocols on how to measure muscle on CT to ensure reproducibility across sites (Láinez Ramos-Bossini et al. 2024). As a result, the practice of arbitrarily selecting cutoff definitions from the literature for individual cohorts should be rendered obsolete.

Our study has some limitations. We included cutoff values at the L3 level only and selected only the most frequently applied cutoff values from the literature. We relied on portal venous–phase CT images for body composition measurements. The degree of enhancement can vary due to patient-related factors and non-patient-related factors, which may reduce the universal reproducibility of the reported attenuation values. Our patient selection was inherently biased by the parent clinical trial (SORAMIC) criteria. All included individuals had unresectable HCC but also met minimum liver function criteria (Child-Pugh A to early B) and performance status requirements. This means extremely frail or sarcopenic patients may have been under-represented, potentially introducing selection bias. Our analysis was confined to a single geographic cohort predominantly from European centers, and we applied a fixed set of literature cutoffs. It is possible that HCC patients in other regions (for example, Asia, where body size and etiologies of HCC differ) might require different muscle mass cutoffs for risk stratification. These limitations notwithstanding, the consistency of our findings—that definition variability alone drastically alters the apparent prognostic value of sarcopenia—is robust and highlights a pressing issue for future research to address.

In conclusion, our study highlights the substantial variability in the prevalence of sarcopenia and myosteatosis, as well as their association with clinical outcomes, depending on the cutoff values applied. This variability underscores a critical challenge in the field of body composition: the lack of standardized, clinically meaningful cutoff points for body composition parameters across different cancer populations. Our findings emphasize the urgent need for establishing standardized cutoff values that are tailored to specific cancer types to improve reliability and applicability of body composition metrics as prognostic tools in oncology. Moving forward, further research should focus on refining image-based cutoff values and validating their prognostic utility across different oncologic settings.

## Data Availability

No datasets were generated or analysed during the current study.

## Declarations

### Guarantor

The scientific guarantor of this study is Alexey Surov.

### Statistics and biometry

One author has significant statistical expertise (Andreas Wienke).

### Study subjects or cohorts overlap

No study subjects or cohorts have been previously reported.
